# Increased male susceptibility to *Mycobacterium tuberculosis* infection is associated with smaller B cell follicles in the lungs

**DOI:** 10.1038/s41598-020-61503-3

**Published:** 2020-03-20

**Authors:** David Hertz, Jannike Dibbern, Lars Eggers, Linda von Borstel, Bianca E. Schneider

**Affiliations:** Junior Research Group Coinfection, Priority Research Area Infections, Research Center Borstel – Leibniz Lung Center, Borstel, Germany

**Keywords:** Tuberculosis, Mucosal immunology

## Abstract

Tuberculosis prevalence is significantly higher among men than women. We have previously revealed an increased susceptibility of male C57BL/6 mice towards *Mycobacterium tuberculosis* (*Mtb*) H37Rv. In the current study, we confirm the male bias for infection with the Beijing strain HN878. Males succumbed to HN878 infection significantly earlier than females. In both models, premature death of males was associated with smaller B cell follicles in the lungs. Analysis of homeostatic chemokines and their receptors revealed differences between H37Rv and HN878 infected animals, indicating different immune requirements for follicle formation in both models. However, expression of IL-23, which is involved in long-term containment of *Mtb* and lymphoid follicle formation, was reduced in male compared to female lungs in both models. Our study reveals sex differences in the formation of B cell follicles in the *Mtb* infected lung and we propose that impaired follicle formation is responsible for accelerated disease progression in males.

## Introduction

Tuberculosis (TB) is the most prevalent bacterial infectious disease in humans. The causative agent, *Mycobacterium tuberculosis* (*Mtb*), is carried by an estimated 2–3 billion people globally and claims approximately 1.5 million lives each year^1^. The global TB pandemic is characterized by significant differences in prevalence between men and women, reflected by a male-to-female ratio for worldwide case notifications of 1.8^1^. Both gender- and sex-related factors likely contribute to higher TB rates in men, but the role of the latter has been largely ignored^2–5^.

We have previously shown an increased susceptibility for male C57BL/6 mice to infection with the laboratory-adapted strain *Mtb* H37Rv^6^. Accelerated disease progression in males after low-dose aerosol infection resulted in increased morbidity and mortality compared to females. Likewise, a study in BALB/c mice revealed that males are more susceptible to H37Rv infection than females^7^. The fact that two of the most widely used TB mouse models do reflect the global male bias in humans emphasizes the need to include both sexes in basic research and pre-clinical studies.

Our observations in H37Rv infected C57BL/6 mice pointed to an impaired formation of lymphoid aggregates in the male lung^6^. In the current study, we substantiate our previous observation and show that B cell follicles that form in the *Mtb* infected lung were much smaller in males compared to females. Moreover, expression of chemokines associated with the homing of lymphocytes to the infected lung such as CXCL13 and CCL19 was significantly lower in males compared to females, further indicating that B cell follicle formation in response to H37Rv infection is impaired in males.

*Mtb* is a member of the *Mtb* complex (Mtbc), and Mtbc strains are more genetically diverse than was previously recognized^8^. Importantly, genetic diversity might contribute to clinical, pathogenic, and immunologic heterogeneity in disease progression and outcome. H37Rv was isolated in 1905 and is not a relevant Mtbc circulating strain today. In contrast, Mtbc strains of the Beijing lineage are emerging worldwide and are associated with the massive spread of multidrug-resistant TB in Eurasia^9^. Clinical isolates of the Beijing lineage are regarded hypervirulent in small animal models due to their rapid growth and reduced survival of infected animals^10–12^. Because it is of major interest to define immune requirements that mediate protective immunity against emerging strains that are of clinical relevance globally we sought to investigate if a male bias was observed after infection of C57BL/6 mice with HN878, the best studied Beijing strain. We herein confirm that the higher male susceptibility in our animal model was independent of the Mtbc strain. In line with our previous observations, premature death of males after HN878 infection was associated with smaller B cell follicles in the lung in the chronic phase of the infection. Analysis of homeostatic chemokines and their receptors revealed differences between H37Rv and HN878 infected animals, indicating different immune requirements for follicle formation in both models. However, expression of IL-23, which is required for long-term control of *Mtb* and B cell follicle formation^13^ was reduced in male compared to female lungs in both infection models.

In conclusion, we show sex differences in the formation of B cell follicles in the *Mtb* infected lung and we propose that impaired follicle formation is responsible for accelerated disease progression in males.

## Methods

### Ethics statement

Animal experiments were in accordance with the German Animal Protection Law and approved by the Ethics Committee for Animal Experiments of the Ministry of Energy, Agriculture, Environment, and Rural Areas of the State of Schleswig-Holstein.

### Mice, bacterial infection and colony forming units (CFU)

C57BL/6 mice were bred under specific-pathogen-free conditions at the Research Center Borstel. Female and male C57BL/6 mice aged 8–12 weeks were used and maintained under specific barrier conditions in BSL 3 facilities. H37Rv and HN878 were grown in Middlebrook 7H9 broth (BD Biosciences) supplemented with 10% v/v OADC (Oleic acid, Albumin, Dextrose, Catalase) enrichment medium (BD Biosciences).

Bacterial aliquots were frozen at −80 °C. Viable cell numbers in thawed aliquots were determined by plating serial dilutions onto Middlebrook 7H11 agar plates supplemented with 10% v/v heat-inactivated bovine serum followed by incubation at 37 °C for 3–4 weeks. For infection of experimental animals, *Mtb* stocks were diluted in sterile distilled water at a concentration providing an uptake of 100 viable bacilli per lung. Infection was performed via the respiratory route by using an aerosol chamber (Glas-Col) as described previously^6^. The uptake was quantified 24 h after infection by determining CFU in the lungs of infected mice. CFU in lung, mediastinal lymph nodes and spleen were evaluated at different time points after aerosol infection by mechanical disruption of the organs in 0.05% v/v Tween 20 in PBS containing a proteinase inhibitor cocktail (Roche) prepared according to the manufacturer’s instructions. Tenfold serial dilutions of organ homogenates in sterile water/1% v/v Tween 80/1% w/v albumin were plated onto Middlebrook 7H11 agar plates supplemented with 10% v/v heat-inactivated bovine serum and incubated at 37 °C for 3–4 weeks.

### Clinical score

Disease progression was assessed by applying a clinical score: Animals were scored in terms of activity, body weight, general condition, and motility/behavior. Each of the criteria is assigned score points from 1 to 5 with 1 being the best and 5 the worst. The mean of the score points represents the overall score for an animal. Animals with severe symptoms (reaching a clinical score of ≥3.5) were euthanized to avoid unnecessary suffering, and the time point that followed was denoted the time of death and scored as 4.

### Multiplex cytokine assay

The concentration of IL-23 in lung homogenates was determined by LEGENDplex^TM^ (Mouse Inflammation panel) according to the manufacturer’s protocol.

### RNA isolation, cDNA-synthesis, and quantitative real-time PCR

Total RNA from lung tissue was extracted using TRIzol^®^ reagent (Invitrogen) as recommended by the manufacturer. For quantitative real-time PCR (qRT-PCR), 2000 ng of total RNA were reverse transcribed using Maxima First Strand cDNA Synthesis Kit for RT-qPCR (Thermo Fisher Scientific) as previously described^14^. Primer sequences are depicted in Table 1.

**Table 1:** List of primer sequences.

IL17A fw	TCTCCACCGCAATGAAGACC
IL17A rev	CACACCCACCAGCATCTTCT
IL23 fw	AATGTGCCCCGTATCCAGTG
IL23 rev	GGAGGTGTGAAGTTGCTCCA
IL-1β fw	ATCAACCAACAAGTGATATTCTCCAT
IL-1β rev	GGGTGTGCCGTCTTTCATTAC
IL-1α fw	CGCTTGAGTCGGCAAAGAAATC
IL-1α rev	GTGCAAGTCTCATGAAGTGAGC
CXCL13 fw	CTCTCCAGGCCACGGTATTC
CXCL13 rev	TTGGCACGAGGATTCACACA
CXCR5 fw	CCCACTAACCCTGGACATGG
CXCR5 rev	ATGTTTCCCATCATACCCAGGAG
CCL19 fw	GTGCCTGCTGTTGTGTTCAC
CCL19 rev	CTTGGCTGGGTTAGGTCTGG
CCL21 fw	CATCCCGGCAATCCTGTTCT
CCL21 rev	CCTCTTGAGGGCTGTGTCTG
CCR7 fw	CCTTGTCATTTTCCAGGTGTGC
CCR7 rev	CCCACGAAGCAGATGACAGA
ICAM-1 fw	GGGACCACGGAGCCAATT
ICAM-1 rev	CTCGGAGACATTAGAGAACAATGC

### Histology

Superior lung lobes from infected mice were fixed with 4% w/v paraformaldehyde (PFA) for 24 h, embedded in paraffin, and sectioned (4 μm). Sections were stained with hematoxylin and eosin (H&E) to assess overall tissue pathology. Ziehl-Neelsen (ZN) method of acid fast staining was used to stain *Mtb* in the lungs (carbol fuchsin (Merck) staining followed by decolorization with acid-alcohol). B cells were detected by anti-B220 (BD Biosciences) and T cells by anti-CD3 (abcam) followed by secondary antibody (biotinylated goat anti rabbit; Dianova), amplification (avidin-HRP) and color reaction (DAB solution; Vectastain). Slides were imaged with a BX41 light microscope and cell^B software. The quantitative analysis of lung size and lymphoid aggregates was conducted using the software ImageJ (freeware). DAB color was deconvolved by the color deconvolution plugin and the threshold was set for the DAB component to exclude unspecific signals. Lung area as well as the number of B220 positive structures regarded as lymphoid aggregates (defined as size >0.01 mm^2^) of the representative images were determined.

### Statistical analysis

All data were analyzed using GraphPad Prism 5 (GraphPad Software, Inc.). Outliers were identified by Grubbs Outlier test with an α-value ≤ 0.05. Statistical analysis was performed by unpaired Student’s t-test or log rank test as described in the figure legends. Values of *p ≤ 0.05, **p ≤ 0.01 and ***p ≤ 0.001 were considered significant.

## Results

### *Mtb* H37Rv infection in males is associated with smaller B cell follicles

**Figure 1:**
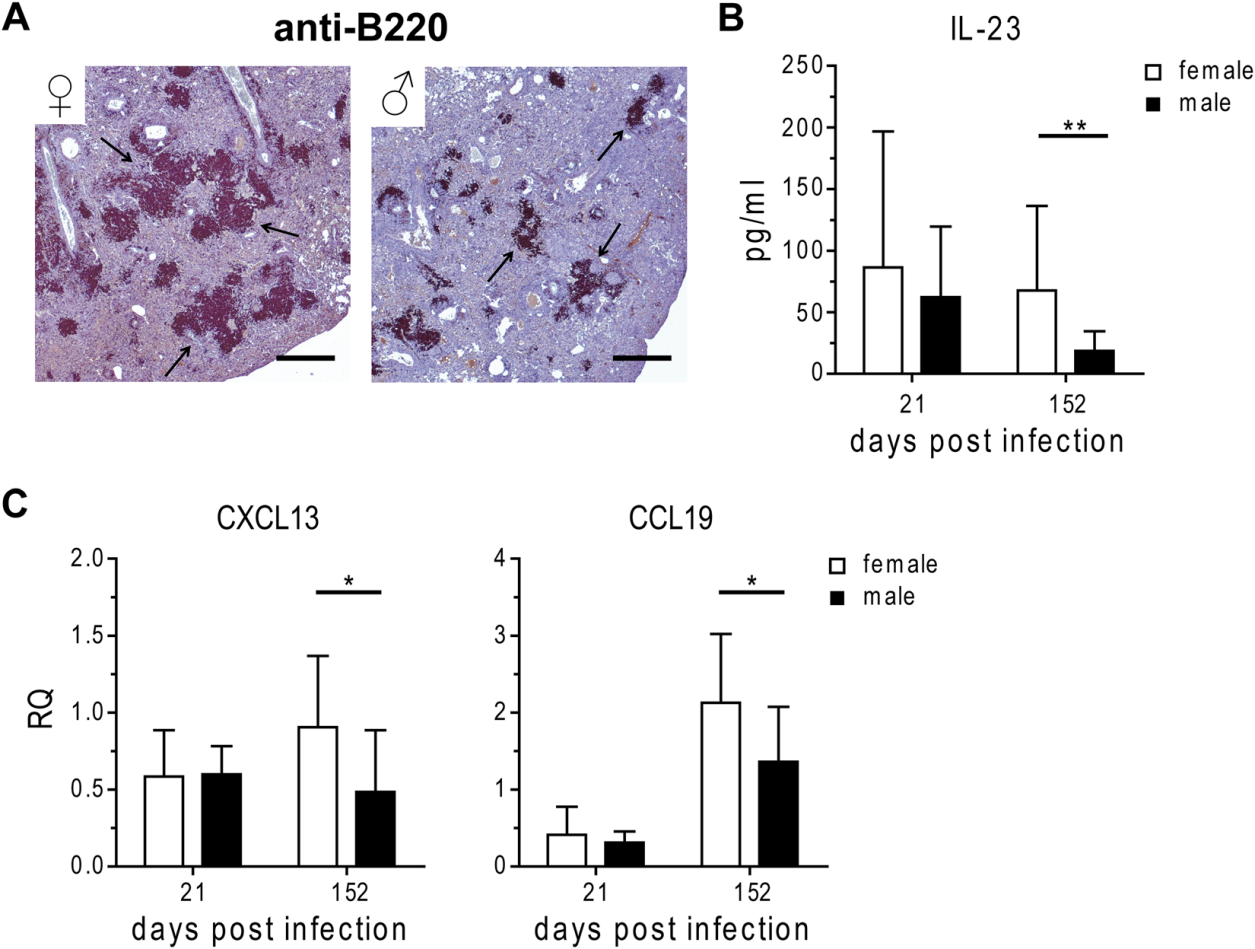
Reduced cytokine and chemokine expression associated with lymphoid follicle formation in male H37Rv infected mice. Females and males were infected via aerosol with H37Rv and lungs were collected at day 152 post infection for (**A**) anti-B220 staining of PFA-fixed, paraffin-embedded lung tissue sections. Arrows represent B220 positive follicles. Representative micrographs from one mouse out of ten mice per group are shown. Bar = 500 µm. In addition, lungs were collected at day 21 and day 152 post infection for analysis of (**B**) protein expression of IL-23 and (**C**) expression of chemokines associated with formation of lymphoid aggregates by qRT-PCR (relative expression (RQ) to housekeeping gene HPRT). Data is presented as mean ± SD (n = 10). Statistical analysis was performed by Student’s t-test. *p ≤ 0.05, **p ≤ 0.01.

We previously showed that a reduced resistance of male C57BL/6 mice to aerosol infection with H37Rv was associated with smaller lymphoid aggregates in the infected lung^6^. Immunohistochemical evaluation of lung sections revealed that lymphoid aggregates are rich in B220 positive B cells (Fig. 1A), reminiscent of ectopic lymphoid structures (ELS) which resemble secondary lymphoid organs in structure and cell composition. ELS are characteristic of both human and mouse granulomas, and associated with immune control during TB^15–17^. Importantly, while female lesions featured large B220 positive follicles these appeared much smaller in males (Fig. 1A, arrows), indicating that increased male susceptibility is associated with defective lymphoid follicle formation.

The development and organization of ELS requires homeostatic chemokines and inflammatory cytokines. Several of these mediators are required for long-term control of *Mtb* infection. For example, IL-23 is required for long-term control of *Mtb* and expression of CXCL13, which instructs B cell follicle formation and maintenance^13,18^. Of note, IL-23 was significantly reduced in males shortly before their condition began to deteriorate (Fig. 1B)^6^. In good agreement, the expression of CXCL13 was significantly lower in male compared to female lungs at the same time point (Fig. 1C). Moreover, CCL19, which recruits CCR7-expressing dendritic cells (DCs) and T cells to ELS, was expressed at a significantly lower level in male compared to female lungs infected with H37Rv (Fig. 1C).

In conclusion, smaller B cell follicles together with reduced expression of IL-23, CXCL13 and CCL19 indicate that ELS formation in response to H37Rv infection is impaired in the male lung.

### Increased susceptibility of male C57BL/6 to *Mtb* HN878 infection

**Figure 2:**
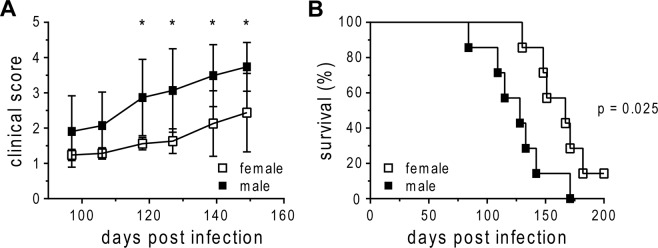
Increased susceptibility of male C57BL/6 mice to HN878 infection. Females and males were infected via aerosol with a low dose of HN878 and monitored for onset of clinical signs (**A**) and survival (**B**). For clinical score dead mice were scored as 4. Data from one experiment out of two are presented as mean ± SD (**A**; n = 7) or each data point represents one mouse (**B**). Statistical analysis was performed by Student’s t-test (clinical score) or log rank test (survival). *p ≤ 0.05.

To investigate if males were also more susceptible to infection with a more relevant clinical strain, we studied the outcome of low dose aerosol infection with the Beijing clinical reference strain HN878 in C57BL/6 mice. Disease progression in males was accelerated compared with females, as reflected by an earlier onset of clinical symptoms which resulted in a significantly increased clinical score (Fig. 2A). Consequently, males succumbed to HN878 infection significantly earlier than females (Fig. 2B).

**Figure 3:**
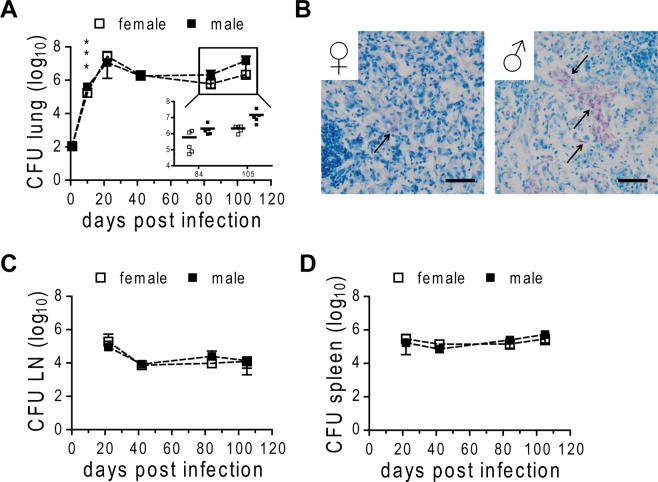
Bacterial burden in HN878 infected male and female C57BL/6 mice. Females and males were infected via aerosol with a low dose of HN878. CFU were determined in homogenates of lung (**A**), lymph node (LN; **C**), and spleen (**D**) at different time points. (**B**) PFA-fixed, paraffin-embedded lung tissue sections from day 105 post infection were subjected to ZN staining for detection of HN878 (arrows). Representative micrographs from one mouse out of five mice per group are shown. Bar = 50 µm. (**A**,**C** and **D**) Data is presented as mean ± SD (n = 5). Statistical analysis was performed by Student’s t-test. ***p ≤ 0.001.

We next compared the lung bacterial burden in males and females at different time points following HN878 infection. There was a small increase in bacterial numbers in males very early after infection (day 10; Fig. 3A), however as mice entered the chronic phase, the bacterial burden was controlled on comparable levels in both sexes. Interestingly, mycobacterial loads started to diverge in the later stage of infection, with 0.5–1 log unit difference in CFU between the sexes on day 105, indicating reduced control over bacterial replication in males compared to females (Fig. 3A, insert). To substantiate our observation, we evaluated HN878 loads in the lung on paraffin-embedded tissue sections by ZN staining. High numbers of mycobacteria were detected in male lungs, which frequently harbored large clusters of acid-fast bacilli in contrast to females (Fig. 3B, arrows). We did not observe differences in bacterial loads in the mediastinal lymph nodes and spleen between the sexes (Fig. 3C,D) suggesting that bacterial dissemination from the lungs to other organs was not enhanced in males.

### Impaired B cell follicle formation in males in response to HN878 infection

**Figure 4:**
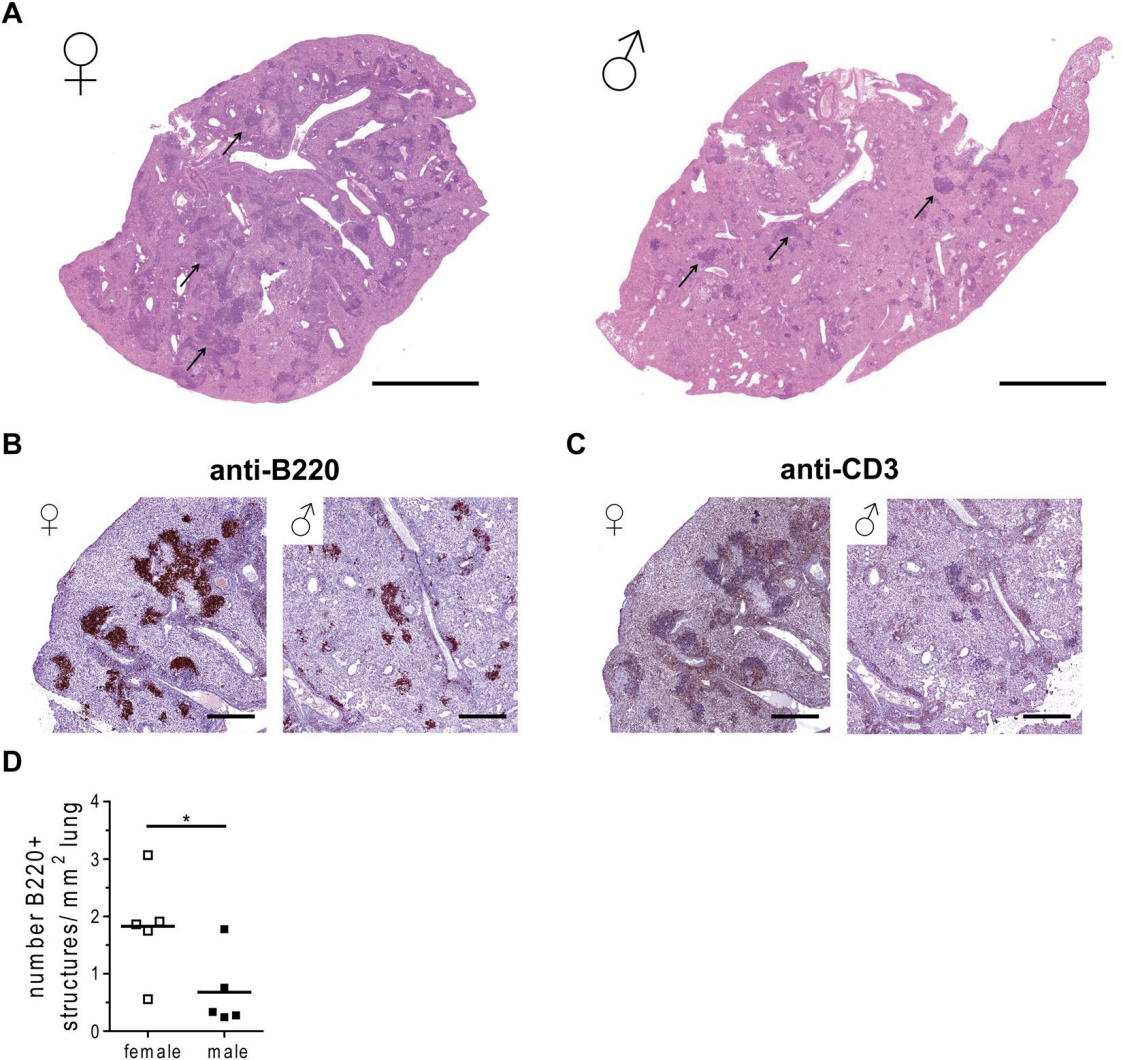
Reduced number of lymphoid aggregates in *Mtb* infected male C57BL/6 mice. Females and males were infected via aerosol with a low dose of HN878. Lungs were collected at day 105 and PFA-fixed, paraffin-embedded tissue sections were stained with H&E (**A**; arrows: lymphoid aggregates), or stained with antibodies to detect (**B**) B220, or (**C**) CD3. Representative micrographs from one mouse out of five mice per group are shown. Bar = 2 mm (**A**) and 500 µm (B + C). (**D**) Quantitative analysis of the number of B220 positive structures shown in (**B**) per mm^2^ lung tissue from 5 mice per group. Each data point represents one mouse. Statistical analysis was performed by Student’s t-test. *p ≤ 0.05.

Our previous investigations revealed that lymphoid aggregates were much smaller in H37Rv infected male compared to female lungs^6^ (Fig. 1). Histological analysis of late-stage HN878 infected lungs also revealed striking differences in the organization of the granulomatous lesions between the sexes (Fig. 4A, arrows). Lymphoid aggregates were rich in B220 positive B cells and associated with CD3 positive T cells in both sexes (Fig. 4B,C). However, males exhibited a strong decrease in B220 staining compared to females (Fig. 4B). Consequently, quantitative analyses of B220 positive structures confirmed lower numbers of B220 positive follicles per mm^2^ lung in males compared with females (Fig. 4D).

### Cytokine responses involved in lymphoid follicle formation differ between the sexes

**Figure 5:**
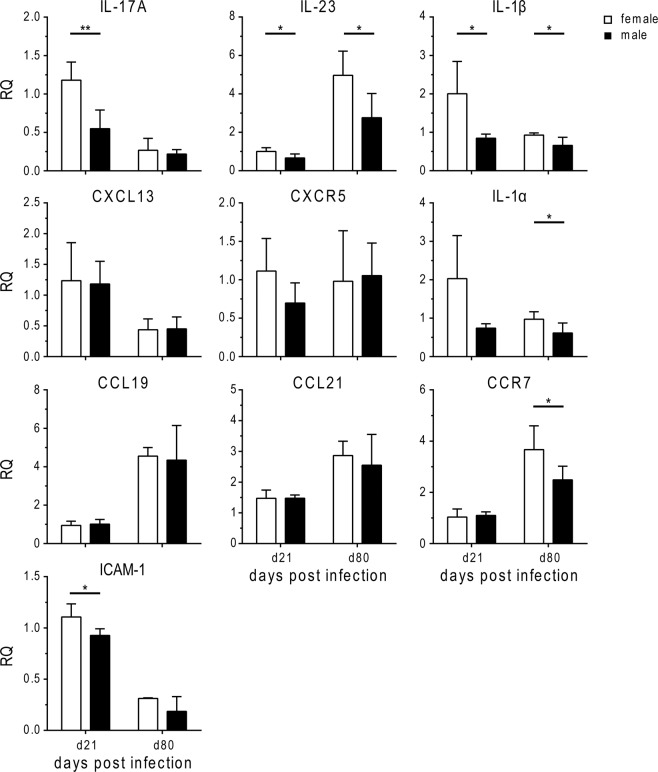
Expression of cyto- and chemokines involved in lymphoid follicle formation. Females and males were infected via aerosol with a low dose of HN878. Lungs were collected at day 21 or day 80 after infection for analysis of expression of typical mediators associated with formation of lymphoid aggregates by qRT-PCR (relative expression (RQ) to housekeeping gene HPRT). Data are presented as mean ± SD (n = 5). Statistical analysis was performed by Student’s t-test. **p ≤ 0.01; *p ≤ 0.05.

In contrast to H37Rv, HN878 induces a strong Th17 response and stimulates IL-17A expression via TLR-2 dependent IL-1β production^19^. Importantly, IL-17A contributes to protective immunity against HN878 and to the development of ELS^19–23^. Therefore, we sought to determine IL-17A expression during HN878 infection. Indeed, IL-17A, which was hardly detectable in H37Rv infected mice (data not shown) was readily induced by HN878, but IL-17A expression was significantly lower in males compared to females early during infection (Fig. 5). In addition, the expression of IL-23 and IL-1β which act upstream of IL-17A^24^ was significantly decreased in males compared to females both in the early and late stage of HN878 infection (Fig. 5). Likewise, IL-1α expression was reduced in males at both time points. To our surprise, despite significantly smaller B cell follicles and reduced expression of critical cytokines in male lungs, we did not detect differences in the expression of CXCL13 or its receptor CXCR5 between the sexes at the time points analyzed (Fig. 5). Moreover, expression of homeostatic chemokines CCL19 and CCL21 did not differ between HN878 infected males and females either. Instead, transcripts for CCR7, the receptor for CCL19 and CCL21, were significantly lower in males compared to females in the late stage of HN878 infection, indicating reduced recruitment of DCs and/or T cells to the site of infection. In addition, expression of ICAM-1, which is required for the migration of leukocytes from the blood into infected tissue, was significantly lower in male compared to female lungs early after HN878 infection.

In conclusion, our data strongly suggest that immune mechanisms required for the generation of B cell follicles during *Mtb* infection are impaired in males. Reduced B cell follicles may contribute to the reduced resistance and premature death observed in males.

## Discussion

Next to socioeconomic and cultural factors, the biological sex significantly contributes to differences between the sexes in infectious diseases. We have previously shown that male C57BL/6 mice are more susceptible to H37Rv infection^6^. Together with the data presented here our observations in H37Rv-infected C57BL/6 mice point to an impaired formation of ELS in the male lung. These structures likely contribute significantly to the host response to *Mtb* as they orchestrate the local host defense and enable highly efficient interaction among the various immune cells such as DCs, macrophages and T cells present in the granulomatous tissues^13,16–18,25^. The B cell chemoattractant CXCL13 is strongly associated with lymphoid neogenesis and orchestrates the homing of CXCR5 expressing lymphocytes to the follicular areas^17,18,26,27^. Lack of CXCL13 compromises immunity to TB, which is linked to poor follicle formation and T cell recruitment from the vessels into the lung parenchyma^13,18^. Likewise, the homeostatic chemokines CCL19 and CCL21 are required for the organization of lymphoid areas in the *Mtb*-infected lung^13,18,28^. In the current study, we found reduced expression of CXCL13 and CCL19 in males chronically infected with H37Rv. In good agreement with reduced chemokine expression, B cell follicles were smaller in size in males at a time point when they started to show first clinical symptoms^6^, indicating that impaired lymphoid follicle formation in the lungs facilitate disease progression. Importantly, IL-23 which mediates long-term control of *Mtb* and B cell follicle formation via induction of CXCL13^13^ was significantly decreased in male compared to female lungs. Together, our study reveals that the male sex is associated with impaired production of cyto- and chemokines that mediate long-term control of H37Rv via the instruction of ELS.

The human-adapted members of the Mtbc comprise seven phylogenetic lineages that differ in their geographical distribution. There is growing evidence that this phylogeographic diversity modulates the outcome of TB infection and disease^29^ and many studies have demonstrated that the bacterial genotype influences virulence and immunogenicity in experimental models. Therefore, we wondered whether we could validate our findings with H37Rv in a different genetic background. HN878 is a representative of the Beijing family of strains and shows an increased virulence in small animal models^10–12^. In good agreement with previous reports, C57BL/6 mice succumbed to HN878 infection much earlier compared to H37Rv infection in the present study. Importantly, we show for the first time that males died significantly earlier than females, confirming the male bias observed after H37Rv infection for HN878. Histological analysis of HN878 infected lungs revealed smaller lymphoid structures in males similar to what we observed during H37Rv infection. B cell rich follicles and associated CD3 positive T cell zones were significantly diminished in the male lesions, indicative of defective formation of ELS as observed before in H37Rv infected males.

In order to get an idea of sex differences in immune regulation during HN878 infection we analysed the expression of a number of cyto- and chemokines known to be involved in ELS formation. None of the mediators we analysed was differently expressed in naïve males and females except for CCL21 which was significantly higher in naïve males (data not shown). In HN878 infected mice, we observed a clear sex difference in the expression of IL-17A, IL-23 and IL-1β, which was significantly higher in female compared to male lungs. While expression of IL-17A and IL-1β was highest early after infection, expression of IL-23 was elevated in the late stage of HN878 infection when differences in susceptibility between the sexes became apparent. Interestingly, CCR7, which can mediate IL-23 production in murine DCs^30^ followed a similar expression pattern, with higher levels in female compared to male lungs in late stage infection. CCR7 is relevant for B-cell follicular development in the lungs. Its absence led to disturbed B cell follicle formation during H37Rv infection^28^. While susceptibility of CCR7-deficient mice to low-dose H37Rv infection was not increased, CCR7 knockout mice died significantly earlier than WT mice after infection with a higher inoculum of H37Rv^31^. This indicates that optimal protective immunity requires CCR7-dependent cell recruitment, which might be of particular importance in infection with more virulent strains such as HN878.

A number of studies have demonstrated differences in innate and adaptive immunological outcomes as a consequence of Mtbc genetic diversity^32^. Intriguingly, our study suggests that despite differences in virulence and immunogenicity between H37Rv and HN878, impaired B cell follicle formation is a common feature of higher susceptibility in males. Reduced IL-23 expression was consistently observed in males in both infection models. It was previously shown that IL-23 mediated long-term control of H37Rv infection mostly independent of IL-17A^13^. Thus, the sex-dependent role of IL-23 and downstream cytokines in both models needs to be further explored. Importantly, androgen receptor signaling was shown to decrease IL-17A and IL-23R expression in Th17 cells^33,34^ while estrogen and progesterone increased IL-23/IL-23R signaling and IL-17A production^35^, raising the possibility that both cytokines are regulated in a sex-hormone dependent manner during *Mtb* infection.

Identifying common denominators of male susceptibility for different members of the Mtbc may facilitate the development of novel therapeutic or preventive approaches that restore clinically-relevant immune responses in a sex-specific manner. Of note, lymphoid follicles not only form during *Mtb* infection but also upon mucosal vaccination, which induces superior protection against TB in animal models^36–39^. These vaccination studies suggest that the interplay between B and T cells is critical for the proper development of protective immune responses to TB and corroborate previous studies which demonstrated the importance of B cells for control of TB^40–42^. In good agreement, a very recent report showed that HN878 infection resulted in increased bacterial loads and increased inflammation in lungs of B-cell deficient mice^43^. In latent TB in humans, B cells contributed to the production of multiple cytokines such as IL-1β, IL-17A, IL-21 and IL-10^44^ and the antibody profiles of persons with latent TB differed from those observed in individuals with active TB^45^. Moreover, B-cell dysfunction compromised cellular host immunity during *Mtb* infection^46^ while ectopic lung B cell formation was associated with containment of *Mtb*^47^. Together, B cells presumably have a greater role in the host defense against *Mtb* than previously thought.

Our study showed smaller B cell follicles in males exhibiting greater susceptibility to TB, and point to a putative role for IL-23 in these dimorphisms. We hypothesize that impaired formation of B cell follicles not only promotes disease progression during *Mtb* infection but also affects vaccine efficacy in males. Thus, understanding differences in B cell follicle formation between males and females may facilitate the development of individualized vaccination approaches, which will be critical to protect both sexes against *Mtb* infection in the future.
